# Co-ensiling pomegranate (*Punica granatum* L.) peels and molasses with berseem (*Trifolium alexandrinum* L.) alters fermentation quality, nutrient composition, ruminal fermentation and methane production in buffalo bulls *in-vitro*

**DOI:** 10.1007/s11250-024-04259-6

**Published:** 2025-01-09

**Authors:** Mariam G. Ahmed, Samir Z. El-Zarkouny, Adham A. Al-Sagheer, Eman A. Elwakeel

**Affiliations:** 1 Animal Production Research Institute, Agricultural Research Center, Nadi El-Said, Giza, 11622 Egypt; 2 Department of Animal and Fish Production, Faculty of Agriculture (El-Shatby), Alexandria University, Alexandria, 21545 Egypt; 3 Department of Animal Production, Faculty of Agriculture, Zagazig University, Zagazig, 44511 Egypt

**Keywords:** Pomegranate peels, Methane production, Legume forages, Silage, Rumen fermentation

## Abstract

Pomegranate peels are an industrial by-product high in sugar and phytochemical content and pose an environmental concern. Meanwhile, ensiling legume forage such as berseem is difficult due to its lower dry matter content and water-soluble carbohydrate-to-buffering capacity ratio, which leads to a poor fermentation process. To date, no studies have been conducted to investigate the effect of co-ensiling pomegranate peels with berseem. Thus, silage quality was evaluated after co-ensiling of berseem (control) with 50, 100, and 200 g/kg pomegranate peels or 50 g/kg molasses for 0, 15, 30, and 45 days (Experiment 1). Further, rumen nutrient degradation, methane production, and rumen fermentation parameters were evaluated in vitro (Experiment 2). Pomegranate peels (200 g/kg) and molasses reduced silage pH compared to control (4.41 or 3.79 vs. 5.02), ammonia-N (2.66 or 3.14 vs. 13.39 g/kg N), and butyric acid (0.05 or 0.1 vs. 0.96 g/kg DM) however, dry matter (323.5 or 283.6 vs. 212.8) and non-fiber carbohydrates (264.8 or 351.8 vs.136.9 g/kg) were increased, respectively. Pomegranate peels and molasses significantly (*P* < 0.05) increased rumen nutrient degradation and significantly (*P* < 0.05) decreased methane and ammonia-N production (Experiment 2). The chemical composition of silage, in-vitro rumen fermentation, and silage quality parameters were significantly (*P* < 0.05) correlated. Pomegranate peels and molasses have potentially improved silage quality and positively influenced rumen fermentation parameters.

## Introduction

Berseem (*Trifolium alexandrinum L.)* is an excellent legume forage extensively utilized in ruminant diets as hay and silage (Ahmed et al. 2023; Yuan et al. 2017). Despite its nutritional advantages, berseem faces challenges related to proteolysis during ensiling and ruminal digestion, leading to an underestimation of its protein value (Barros et al. 2017). This degradation process affects silage quality by increasing buffering capacity, hindering the achievement of low pH levels, and promoting undesirable clostridial fermentation, resulting in poor fermentation quality of silage (Kung et al. 2018). Meanwhile, poor silage quality leads to elevated rumen methane (CH_4_) emissions, which subsequently cause a 2–12% decrease in gross energy availability for the host ruminant animal and reduced feed utilization efficiency (Evans 2018). As a result, there is a worldwide need to improve legume forage ensiling techniques to improve silage quality, preserve nutritional value, and reduce environmental impact (Ahmed et al. 2023; Chen et al. 2021). Molasses is a byproduct of sugarcane, sugar beet, starch, citrus, and hemicellulose. It is composed of 79% soluble carbohydrates and 45–50% sucrose. Molasses, a common fermentation stimulant, is employed to promote the growth of lactic acid bacteria in legume forage silage (Gao et al. 2021). However, molasses’s high viscosity makes it difficult to handle and does not affect animal performance (Oladosu et al. 2016).

Pomegranate (*Punica granatum* L.) is a member of the Punicaceae family and is extensively grown in different areas including Asia, Africa, and Europe. It is commonly regarded as a functional fruit due to its health-promoting benefits (Ahmed et al. 2022; Karimi et al. 2017). When pomegranates are industrially processed into juice, by-products such as the peel account for 40 to 60% of the weight of the fruit (Natalello et al. 2020). Pomegranate peels are high in botanical compounds such as flavonoids, anthocyanidins, tannins, and readily fermentable soluble sugars (e.g., pectin) (Natalello et al. 2020; Niu et al. 2023). Pomegranate peels have recently been used as natural antibiotic alternatives in ruminant diets to improve animal productivity and control rumen fermentation (Niu et al. 2023). Although ensiling pomegranate peels have demonstrated acceptability and safety in animal feed (Khorsandi et al. 2019), further research is warranted on their utilization to enhance the quality of legume forage fermentation.

To our knowledge, no studies have been conducted to investigate the effect of co-ensiling pomegranate peels with berseem. Given the advantageous properties outlined above, it is hypothesized that the co-ensiling of pomegranate peels with berseem could potentially enhance the chemical composition and fermentation quality of the silage, while also positively manipulating rumen fermentation. Therefore, this study aimed to assess the impact of ensiling dried pomegranate peels or molasses on the fermentation quality and chemical composition of berseem silage across various ensiling durations. Additionally, the study sought to evaluate rumen nutrient degradation, methane production, and in vitro rumen fermentation parameters.

## Materials and methods

### Experiment 1: evaluation of silage quality

#### Raw materials and silage preparation

The pomegranate peels were acquired from a food factory in Alexandria’s Borg El-Arab industrial zone. Peels were then dried at 50°C in a forced oven for 72 h. Then, the peels were pulverized using (Arthur H. Thomas, Philadelphia, PA, USA) until they could pass through a screen with a 1 mm. Molasses was purchased from Harvest Foods Company (Borg El-Arab, Alexandria). Berseem (*Trifolium alexandrinum* L.) was harvested as a fourth cut from a private farm located in the Abees region of Alexandria city, Egypt. Following a 24-h wilting period, the berseem was chopped by a hand cutter into pieces measuring 15–20 mm in length. Chopped forage was individually ensiled with 0, 50, 100, or 200 g/kg dried pomegranate peels or 50 g/kg molasses (on a fresh matter basis). Then, the ingredient mixture was compressed, packed in a mini plastic silo (9 cm diameter × 16 cm height) with a plastic lid. The silo had a capacity of 1 kg. After 0, 15, 30, and 45 days, 60 silos (4 sampling times × 5 treatments × 3 replicates) were opened and maintained at the ambient temperature (20–22ºC).

#### Evaluation of silage fermentation criteria

The contents of each silo from each treatment were independently evaluated at the end of each period of ensiling to assess the quality of fermentation. Silage samples weighing 20 g were homogenized in 100 mL of distilled water for one minute, allowed to stand at room temperature for one hour, and subsequently filtered using four layers of gauze. The filtered liquid was tested for pH, ethanol, volatile fatty acids (VFA), lactic acid, and ammonia-N (NH_3_-N) concentrations. A pH meter (Adwa AD 11 waterproof; Szeged-Hungary Europe, Romania) was used to measure the pH. Lactic acid was measured following Borshchevskaya et al. (2016) procedure. The filtered liquid was prepared in the same manner that Ahmed et al. (2023) carried out to determine the concentrations of VFA, ethanol, and NH_3_-N. Fleig’s score (FS) was calculated using the following equation, according to Kilic (1986):

1 $$\text{Fliegss score}=220+(2\times\%\mathrm{DM}-15)-40\times\text{pH}$$

Based on this index, a score of 81–100 signifies very good quality, a score of 61–80 signifies good quality, a score of 41–60 signifies satisfactory quality, a score of 21–40 signifies medium quality and a score of 20 or lower signifies bad quality.

About 100 g of silage was withheld and dried at a temperature of 50ºC for 72 h in an oven with forced air circulation for the determination of dry matter (DM). Then samples were pulverized using a screen with a mesh size of 1 mm. Organic matter (OM), ash, ether extract (EE), and crude protein (CP) percentages were determined using the methodology outlined by the AOAC (2006). Acid detergent lignin (ADL), neutral detergent fiber (NDF), and acid detergent fiber (ADF) were determined using an ANKOM 220 fiber analyzer (ANKOM, model A2001, Macedon, NY, USA) as described by Van Soest et al. (1991) without the use of sodium sulfite and heat stable amylase. The calculation of non-fiber carbohydrates (NFC) involved subtracting the sum of %NDF, %CP, %EE, and ash% from 100%. The condensed tannins (CT) total tannins (TT), and total phenols (TP) in the raw materials were also estimated (Makkar et al. 1993). The aluminum chloride colorimetric method was used to measure total flavonoids (TF), as reported by Zarina and Tan (2013).

### Experiment 2: in vitro ruminal fermentation of silage

#### Experimental design, inoculum donor, and incubation system

In vitro ruminal fermentation of the silage was measured only after 30 days of ensiling, as this period achieved the highest Flieg’s score, indicating the best silage quality. Following this period, a total of 200 g from each of the 15 mini-silos were opened, thoroughly mixed, and subjected to drying in an air-forced oven at a temperature of 50°C for a duration of 72 h. Subsequently, the dried contents were finely crushed to a particle size of 1 mm, to be used later as feed substrates. Following the protocol established by Menke and Steingass (1988), 500 mg of dried silage samples were placed into a 120 mL serum bottle for in vitro ruminal batch culture examination.

The rumen fluid used in the study was sourced from two buffalo bulls that were slaughtered at the Agricultural Experimental Station of the Agriculture Faculty (El-Shatby), University of Alexandria, Egypt. The bulls had an average body weight of 500 ± 25 kg. The male buffalo were provided with a primary diet consisting of commercial concentrate (14% CP) and rice straw. Collecting ruminal material from slaughtered animals aligns with animal welfare rules and reduces the stress experienced by live animals requiring a surgical procedure (Mutimura et al. 2013). Rumen contents were individually collected immediately after slaughtering in a tank that had been warmed in advance to a temperature of 39°C. The collected contents were subsequently transported to the laboratory within a time frame of 15 min.

In the laboratory, the ruminal contents from each animal were individually strained through four layers of muslin. The strained contents were then placed on a magnetic stirrer at a temperature of 39°C and flushed with CO_2_. Ruminal fluid and McDougall’s buffer were blended in a 1:2 v/v ratio and added to each serum bottle containing 50 mL of inoculation medium. This process was done while continuously flushing the bottles with CO_2_. Serum bottles were filled with buffered rumen fluid, but no substrate was used as a blank in each inoculum. For each treatment, duplicate fermentations for each of the two inoculums (sources) were inoculated under anaerobic conditions. Two separate sets of serum bottles containing the inoculum were sealed with butyl rubber stoppers and aluminium crimps. The first set was then incubated at 39°C for 24 h, while the second set was left to incubate for 48 h. The total number of bottles per run was 48 bottles [(blank + 5 treatments) × 4 bottles × 2 time points (24 and 48 h)]. Each parameter was represented by 8 replicates per treatment (4 replicates × 2 runs).

#### Sample collection and measurements

The same serum bottles (*n* = 8 per treatment) used for gas production measurements were also utilized for estimating the degradability of DM, OM, and NDF and fermentation parameters at each time point (24 and 48 h of incubation). One millimeter of fermentation liquid was collected and centrifuged at 30,000 g for 15 min at 4°C to determine lactate, NH_3_-N, and VFA concentrations according to “Evaluation of silage fermentation criteria” section. The remnants in the bottle were strained using pre-weighed crucibles and rinsed with distilled water. To estimate the apparent DM deterioration (ADMD), the crucibles were dried at a temperature of 110°C for 24 h. ADMD was determined by subtracting the weight of filled crucibles from the weight of empty crucibles and adjusting for any residual material present in the blank. The true degradability of DM, OM, and NDF in the dried residue was estimated as described by Ahmed et al. (2023). Using the equation developed by Czerkawski (1986), the amount of microbial nitrogen (MN) was determined as follows;

2 $$\text{MN }(\text{g}/\text{kg OMD})=\text{OMD}\times 19.3$$

Additionally, methane (CH_4_) production was calculated using the formula equation according to Williams et al. (2019):

3 $$\text{Methane yield }(\text{g}/\text{kg DM})=4.08\times (\text{acetate}/\text{propionate})+7.05.$$

#### Statistical analysis

Data analysis was conducted using the mixed procedure of SAS (version 9.0, SAS Institute Inc., Cary, NC, USA). In Experiment 1. The model incorporated fixed effects treatment (berseem [control], pomegranate peels [50, 100, and 200g/kg] and molasses [50g/kg]), time (0, 15, 30 and 45 days of ensiling) and treatment × time interaction. To assess the treatment trend, orthogonal contrasts were employed to determine the linear, quadratic, and cubic trends. Proper contrasts were also utilized to compare the impact of molasses vs. control treatments. In Experiment 2, the model incorporated fixed effects of treatment (berseem [control], pomegranate peels [50, 100, and 200g/kg] and molasses [50g/kg]), time (24 and 48 h of incubation) and treatment × time interaction. Rumen fluid donor or replicate (*n* = 8) as blocking factor (block). Linear, quadratic, and cubic trends of treatments were assessed using orthogonal contrasts, and proper contrasts were also utilized to compare the impact of molasses vs. control treatments similar to Exp. 1. For mean separation of treatments, the Duncan multiple range test was applied, and significance was declared at a threshold of *P* < 0.05. Pearson correlation heatmap was done using GraphPad Prism Software (version 8, San Diego, CA, USA) to determine the relationship between silage quality parameters and chemical compositions of berseem silage.

## Results

### Experiment 1

#### Characteristics of raw materials before ensiling

The chemical composition of berseem and dried pomegranate peels is shown in Table 1. Wilted berseem (24 h) and dried pomegranate peels contained 255 and 902.8 g/kg DM, respectively. Dried pomegranate peels contain more EE and NFC than berseem, but less structural carbohydrate and CP. Moreover, the dried pomegranate peels with the highest TP, TT, CT, and TF content were 104.6, 76.9, 1.6, and 3.1 g/kg DM, respectively.

**Table 1:** Chemical composition (g/kg DM) of raw materials before ensiling

Item	Wilted berseem	Pomegranate peels
Dry matter	255.0	902.8
Organic matter	866.4	960.7
Crude protein	172.2	39.1
Ether extract	17.1	31.9
Neutral detergent fiber (NDF)	592.5	282.2
Acid detergent fiber (ADF)	335.6	172.9
Acid detergent lignin (ADL)	66.7	48.2
Cellulose^a^	268.9	124.7
Hemicellulose^b^	256.9	109.3
Non-fiber carbohydrate^c^	83.6	607.5
Total phenol (eq-g tannic acid kg DM)	19.0	104.6
Total tannins (eq-g tannic acid kg DM)	3.0	76.9
Condensed tannins (eq-g leucocyanidin/kg DM)	0.2	1.6
Flavonoids (eq. mg rutin/kg DM)	1.3	3.2

^a^Hemicellulose was calculated as NDF minus ADF

^b^Cellulose was calculated as ADF minus ADL

^c^Non-fiber carbohydrate calculated by difference [1000 − (NDF + CP + EE + ash)]

#### Fermentation quality of dried pomegranate peel silage

The fermentation profile of silages with dried pomegranate peels and molasses addition was significantly influenced by the treatment, ensiling time, and their interaction, as indicated in Table 2 (*P* < 0.01). The pH of the silage decreased linearly (*P* < 0.05) as the proportion of dried pomegranate peels in the mixture increased, and a significant drop in pH was observed at 30 and 45 days of ensiling; pH was less than 4.5 for high mixing proportions of pomegranate peels. Consistent with the declining pH, lactic acid increased linearly (*P* < 0.01), with the highest values occurring at 15 and 30 days of ensiling for all ensiled forages.

**Table 2:** Effect of berseem ensiled with molasses and pomegranate peels at different levels on pH content, organic acids, ethanol, and Fleig’s score after 0, 15, 30, and 45 days of ensiling

Item	Days	Con	Treatment^a^				SEM^b^	Contrast *P*-value^c^			
			Pomegranate peels, g/kg DM								
			50	100	200	Mls		PM-L	PM-Q	PM-C	Mls^d^
pH											
	0	5.59	5.05	4.52	4.42	5.84	0.14	< .01	< .01	< .01	< .01
	15	4.95	4.40	4.40	4.30	3.80	0.11	< .01	0.06	0.14	< .01
	30	4.75	4.30	4.05	4.15	3.75	0.10	0.04	0.83	0.33	< .01
	45	5.02	4.74	4.27	4.41	3.79	0.11	< .01	< .01	< .01	< .01
Lactic (g/kg DM)											
	0	3.91	15.1	26.38	47.5	15.40	2.29	< .01	< .01	< .01	< .01
	15	27.98	55.93	39.35	68.15	52.66	2.69	< .01	0.83	< .01	< .01
	30	33.45	62.65	60.88	50.73	66.8	2.97	0.09	0.01	0.42	< .01
	45	21.27	20.13	40.86	37.33	61.19	2.38	< .01	0.47	< .01	< .01
Acetic acid (g/kg DM)											
	0	1.01	1.78	0.47	0.52	3.55	0.23	< .01	< .01	< .01	< .01
	15	16.91	8.76	5.41	1.96	14.75	1.38	0.66	0.23	< .01	0.38
	30	36.07	21.58	8.59	3.15	17.03	2.68	0.31	0.05	< .01	< .01
	45	26.78	14.10	6.69	1.60	8.14	1.95	< .01	< .01	< .01	< .01
Propionic acid (g/kg DM)											
	0	ND	ND	ND	ND	ND	-	-	-	-	-
	15	0.42	0.02	0.01	0.01	0.01	0.04	0.90	0.68	0.63	< .01
	30	1.17	0.22	0.02	0.00	0.01	0.09	0.90	0.79	0.59	< .01
	45	1.32	0.22	0.04	0.01	0.02	0.11	0.01	0.32	0.07	< .01
Butyric acid (g/kg DM)											
	0	0.00	0.15	0.19	0.23	1.22	0.10	< .01	< .01	< .01	< .01
	15	0.02	0.04	0.05	0.10	0.16	0.01	0.18	0.05	< .01	< .01
	30	0.51	0.09	0.07	0.10	0.15	0.04	0.73	0.67	0.84	< .01
	45	0.96	0.06	0.01	0.05	0.10	0.08	0.90	0.45	0.67	< .01
Ethanol (g/kg DM)											
	0	ND	ND	ND	ND	ND	-	-	-	-	-
	15	2.39	9.74	11.51	14.22	10.92	2.31	< .01	0.02	< .01	0.02
	30	5.26	18.58	16.72	28.95	21.56	4.04	0.07	< .01	< .01	0.08
	45	1.56	1.39	6.62	28.33	7.60	2.21	< .01	0.92	< .01	0.08
Fleig’s score											
	0	15.40	57.83	85.46	102.71	19.74	7.75	0.01	< .01	< .01	0.09
	15	39.29	85.38	71.65	108.49	110.61	5.89	< .01	0.87	0.01	< .01
	30	46.92	85.39	89.57	110.64	114.46	5.44	0.01	0.97	< .01	< .01
	45	30.75	65.40	76.15	93.31	110.12	6.22	< .01	< .01	< .01	< .01

Effects of treatment, time and treatment x time interaction were < 0.01

^a^Con: control: 50, 100 and 200 refer to inclusion of pomegranate peels at 50, 100 and 200 g/kg on fresh weight basis

^b^*SEM* standard error of means

^c^*PM-L* linear effect of pomegranate, *PM-Q* quadratic effect of pomegranate, *PM-C* cubic effect of pomegranate

^d^*Mls* control vs. molasses, *ND* not detected

**Fig. 1:**
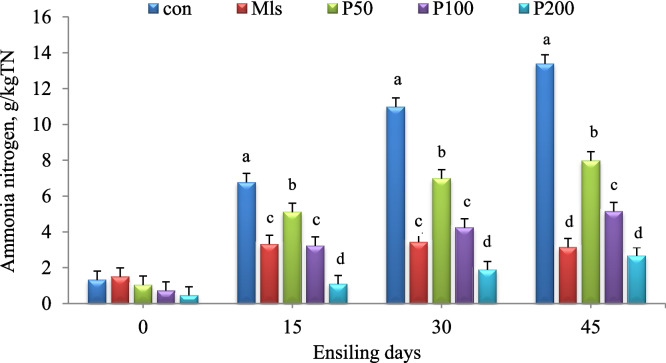
Ammonia-N (g/kg TN) of berseem ensiled with pomegranate peels and molasses at different levels after 0, 15, 30, and 45 days of ensiling. Con: control; Mls: molasses:50,100 and 200 refer to the inclusion of pomegranate peels at 50,100 and 200 g/kg on a fresh weight basis. Effects of treatment, time, and treatment × time interaction are (*P* < 0.01). The standard error of the mean (SEM) is 0.55

At 30 and 45 days of ensiling, increased pomegranate peel content decreased (L,Q,C; *P* < 0.01) acetic acid concentration, with the highest value observed for all ensiled forages after 30 days. After 45 days of ensiling, the amount of propionic acid in mixed silages was also significantly (*P* < 0.05) lower than that of control silages. The response quadratically (*P* < 0.05) of butyric acid and ethanol concentration to increasing pomegranate peel content with a low concentration at an intermediate mixing proportion was observed at 15 and 30 days of ensiling. After 30 days of ensiling, the addition of pomegranate peel to the mixture increased the FS linearly and cubically (*P* < 0.01). The increase in dried pomegranate peel in the mixture decreased cubically in NH_3_-N concentration (*P* < 0.01) and increased significantly as the ensiling progressed (Fig. 1).

At all ensiling times, the molasses treatments had lower pH values and VFA (*P* < 0.01) but higher lactic acid and ethanol content (*P* < 0.01) than the control (Table 2). From days 15 to 45 of ensiling, the NH_3_-N content of molasses treatments was lower (*P* < 0.01) than the control (Fig. 1).

#### Nutrient composition of dried pomegranate peels silage

The chemical composition of berseem silages ensiled with dried pomegranate peels and molasses was significantly influenced by treatment, ensiling time, and their interaction (*P* < 0.01), as indicated in Tables 3 and 4. The content of DM and OM increased (*p* < 0.01; linear, quadratic, and cubic as the proportion of pomegranate peels in the forage mixtures increased. On the contrary, the content of CP in the mixture decreased linearly (*P* < 0.01) with increasing pomegranate peel proportion, with higher values (Q; *P* < 0.01) at intermediate mixing proportions at 45 days of ensiling (Table 3). At 30 days of ensiling, the EE content increased with increasing pomegranate peels in the mixture (*P* < 0.01; linear, quadratic, and cubic). Throughout the ensiling times, the proportion of pomegranate peels in the mixture increased the content of NFC while decreasing the NDF, ADF, hemicelluloses, and cellulose contents of silage (*P* < 0.01; linear, quadratic, and cubic) (Table 4).

**Table 3:** Nutrient composition (g/kg DM) of berseem ensiled with pomegranate peels and molasses and at different levels after 0,15, 30, and 45 days of ensiling

Item	Days	Con	Treatment^a^				SEM^b^	Contrast *P*-value^c^			
			Pomegranate peels g/kg DM								
			50	100	200	Mls		PM-L	PM-Q	PM-C	Mls^d^
Dry matter											
	0	250.0	273.2	306.3	372.6	240.7	1.26	< .01	< .01	< .01	0.45
	15	241.5	281.9	293.3	377.5	288.1	1.17	< .01	< .01	< .01	< .01
	30	239.6	262.0	312.9	358.2	297.3	1.03	< .01	< .01	< .01	< .01
	45	212.8	250.0	288.7	323.5	283.6	0.95	< .01	< .01	< .01	< .01
Organic matter											
	0	866.4	871.1	875.8	885.3	845.9	0.37	< .01	< .01	< .01	0.10
	15	854.7	874.0	884.8	900.8	898.1	0.40	< .01	< .01	< .01	< .01
	30	851.3	869.8	888.2	903.6	891.1	0.43	< .01	< .01	< .01	< .01
	45	858.5	860.1	875.2	895.0	887.7	0.38	< .01	< .01	< .01	< .01
Crude protein											
	0	172.2	165.6	158.9	144.3	164.6	0.26	< .01	< .01	< .01	< .01
	15	154.7	165	145.2	114.1	136.2	0.48	< .01	0.01	< .01	< .01
	30	164.3	146.9	132.2	126	131.4	0.34	< .01	0.02	0.09	< .01
	45	172.1	139.3	154.7	140.1	140.1	0.34	0.02	0.01	0.22	< .01
Ether extract											
	0	17.1	18.9	20.0	23.1	18.4	0.05	0.44	0.51	0.52	0.69
	15	18.2	21.8	22.3	22.7	18.8	0.29	0.33	0.08	0.82	0.06
	30	19.2	21.9	21.7	25.4	19.7	0.29	0.02	< .01	0.03	< .01
	45	19.6	22.0	22.6	24.8	19.7	0.20	0.02	< .01	0.10	< .01

Effects of treatment, time, and treatment × time interaction were (*P* < 0.01)

^a^Con: control: 50, 100, and 200 refer to the inclusion of pomegranate peels at 50, 100, and 200 g/kg on a fresh weight basis

^b^*SEM* standard error of means

^c^*PM-L* linear effect of pomegranate, *PM-Q* quadratic effect of pomegranate, *PM-C* cubic effect of pomegranate

^d^*Mls* control vs. molasses

**Table 4:** Non-fiber carbohydrates and structural carbohydrates compositions (g/kg DM) of berseem ensiled with molasses and pomegranate peels at different levels and ensiled for 0,15, 30, and 45 days

Item^a^	Days	Con	Treatment^b^				SEM^c^	Contrast *P*-value^d^			
			Pomegranate peels, g/kg DM								
			50	100	200	Mls		PM-L	PM-Q	PM-C	Mls^e^
NFC											
	0	83.8	109.9	134.8	187.4	95.3	1.07	< .01	< .01	< .01	< .01
	15	123.9	203.4	241.3	332.5	377.7	2.20	< .01	< .01	< .01	< .01
	30	123.8	205.7	267.8	321.2	359.5	1.88	< .01	< .01	< .01	< .01
	45	136.9	208.1	220.8	264.8	351.7	1.73	< .01	< .01	< .01	< .01
NDF											
	0	593.2	577.7	562.2	530.5	592.5	0.67	< .01	< .01	< .01	0.38
	15	557.9	483.8	475.9	431.5	365.5	1.56	< .01	< .01	< .01	< .01
	30	544	495.3	466.4	430.9	380.5	1.40	< .01	< .01	< .01	< .01
	45	529.9	490.7	477.2	465.4	376.2	1.09	< .01	< .01	0.06	< .01
ADF											
	0	335.7	327.5	319.7	303.1	335.3	0.33	< .01	< .01	< .01	0.33
	15	365.2	304.0	298.9	255.6	229.7	1.17	< .01	< .01	< .01	< .01
	30	363.8	314.2	302.4	264.3	246.8	1.03	< .01	< .01	< .01	< .01
	45	367.6	328.4	308.5	246.5	243.4	1.05	< .01	< .01	< .01	< .01
ADL											
	0	66.7	65.7	64.9	63.0	66.6	0.05	< .01	< .01	< .01	0.22
	15	74.5	64.8	63.1	51.4	46.1	0.29	< .01	< .01	< .01	< .01
	30	70.6	70.9	65.6	56.0	50.3	0.25	< .01	< .01	< .01	< .01
	45	69.6	59.2	69.5	56.9	46.4	0.22	< .01	< .01	< .01	< .01
Cellulose											
	0	269.0	261.8	254.7	240.1	268.7	0.29	< .01	< .01	< .01	0.43
	15	290.7	239.2	235.8	204.3	183.6	0.90	< .01	< .01	< .01	< .01
	30	293.2	243.4	236.8	208.4	196.6	0.83	< .01	< .01	< .01	< .01
	45	298.1	269.2	239.0	189.6	197.0	0.90	< .01	< .01	< .01	< .01
Hemicelluloses											
	0	257.7	250.2	242.5	227.4	257.3	0.34	< .01	< .01	< .01	0.56
	15	192.8	179.8	177.1	175.9	135.8	0.46	< .01	< .01	0.13	< .01
	30	180.2	181.1	164.0	166.7	133.7	0.45	< .01	< .01	< .01	< .01
	45	162.3	162.2	168.7	218.9	132.9	0.57	< .01	< .01	< .01	< .01

Effects of treatment, time, and treatment × time interaction were < 0.01

^a^*NFC* non-fiber carbohydrates, *NDF* Neutral detergent fiber, *ADF* Acid detergent fiber, *ADL* Acid detergent fiber

^b^Con = control: 50, 100, and 200 refer to the inclusion of pomegranate peels at 50, 100, and 200 g/kg on fresh

^c^*SEM* standard error of means

^d^*PM-L* linear effect of pomegranate, *PM-Q* quadratic effect of pomegranate, *PM-C* cubic effect of pomegranate

^e^*Mls* control vs. molasses

The molasses treatment had higher DM, OM, and EE content(*P* < 0.01) but lower CP content (*P* < 0.01) than the control (Table 3). At the ensiling time, the molasses treatment had a higher NFC content (*P* < 0.01) and a lower structural carbohydrate content (*P* < 0.01) than the control. Except for hemicellulose, the silage-treated molasses content increased in NDF, ADF, ADL, and cellulose after 30 and 45 days of ensiling (Table 4).

### Experiment 2. Nutrient degradation, methane production and fermentation profiles

**Fig. 2:**
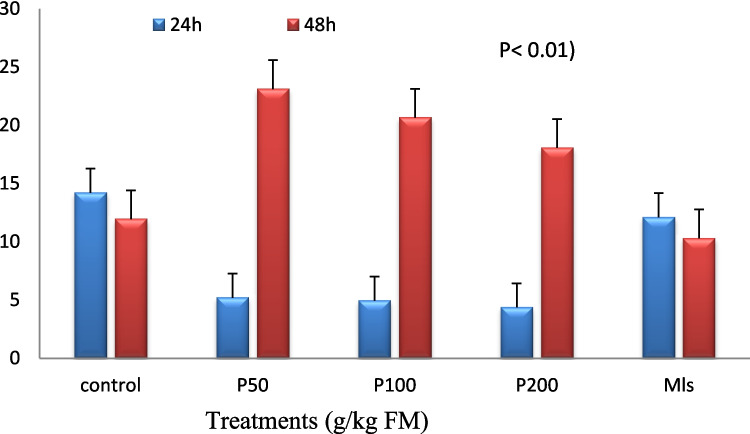
Ammonia -N (mg/100 ml rumen buffered liquid) of berseem ensiled with pomegranate peels and molasses at different levels after 30 days of ensiling. Con: control; Mls: molasses; The p-value for the effect treatment (*P* = 0.6), time (*P* < 0.01), treatment × time (*P* < 0.001). The standard error of the mean (SEM) is 1.08

Overall, there was no significant interaction between treatment and incubation time for nutrient degradation, CH_4_ production, or fermentation patterns. The only interaction between time and treatment on NH_3_-N concentration was significant (*P* < 0.01) (Fig. 2).

The rumen nutrient degradation, methane production, and microbial protein synthesis of pomegranate peels and molasses ensiled with berseem silage are presented in Table 5. As the proportion of pomegranate peels in the mixture increased, the ADMD increased linearly (*P* < 0.001). The TDMD and TOMD exhibited a quadratic response to the increasing pomegranate peel content, with the highest values observed at intermediate pomegranate levels (Q: *P* < 0.05). At high levels of pomegranate peel in the mixture, CH_4_ production decreased cubically (*P* < 0.05). Maximum MN quadratic responses to increasing pomegranate peel content in mixtures (*P* < 0.05). There were no significant differences in lactate, total VFA concentration, or molar proportions of VFA, except for isobutyrate, isovalerate, and Valeric, which decreased (L,Q, C; *P* < 0.05) to increase pomegranate peel content in mixtures while A to P ratio reduced cubically (*P* < 0.05) (Table 6). After 24 h of incubation, the NH_3_-N concentration was lower (*P* < 0.001) at high levels of dried pomegranate peel (Fig. 2).

**Table 5:** Effects of berseem ensiled with molasses and pomegranate peels (ensiled for 30 days) on apparent and true dry matter (ADDM and TDDM), true organic matter (TDOM), neutral detergent fiber disappearance (NDFD), methane (CH_4_) and microbial protein (MP) in vitro

Item	Treatment^a^					SEM^b^	Contrast *P*-value^c^			
	Pomegranate peels, g/kg DM									
	Con	50	100	200	Mls		PM-L	PM-Q	PM-C	Mls^d^
ADDM (%)	54.94	60.87	63.05	52.79	65.33	1.217	< .001	0.32	0.08	< .01
TDMD (%)	65.75	68.58	70.33	62.44	74.08	0.871	< .001	0.06	0.16	< .01
TOMD (%)	62.15	66.11	68.4	58.05	72.21	1.037	< .001	0.04	0.08	< .01
NDFD (%)	33.12	35.47	36.48	34.35	34.92	1.176	0.52	0.96	0.31	0.89
CH_4_ (g/kg DM)	24.26	24.29	24.72	24.11	21.89	0.164	0.10	0.03	0.02	< .01
MN (g/kg of OMD)	118.09	125.61	129.97	110.29	137.2	1.970	< .01	0.02	0.09	< .01

^a^Values are average concentrations at 24 and 48 h of incubation. No treatment × time interaction was observed

^b^Average of SEM among treatments

^c^*PM-L* linear effect of pomegranate, *PM-Q* quadratic effect of pomegranate, *PM-C* cubic effect of pomegranate

^d^*Mls* control vs. molasses

**Table 6:** Effects of berseem ensiled with molasses and pomegranate peels (ensiled for 30 days) on concentrations of short-chain fatty acids (acetic, propionic, isobutyric, butyric, isovaleric, valeric), total VFA, acetate to propionate (A to P) ratio, and lactate in vitro (Exp. 2)

Item	Treatment^a^						Contrast *P*-value^c^			
	Pomegranate peels, g/kg DM									
	Con	50	100	200	Mls	SEM^b^	PM-L	PM-Q	PM-C	Mls^4^
Lactate (mM)	29.31	29.65	28.16	30.22	21.21	1.38	0.94	0.79	0.69	0.16
Total VFA (mM)	115.07	113.54	111.04	112.01	124.68	2.084	0.97	0.93	0.32	0.07
Acetic acid (mM)	77.44	76.36	74.73	74.79	79.58	1.218	0.84	0.80	0.25	0.27
Propionic acid (mM)	18.37	18.08	17.25	17.94	21.85	0.382	0.78	0.70	0.10	0.01
Butyric acid (mM)	13.44	13.57	14.02	12.73	17.11	0.447	0.44	0.49	0.80	0.02
Isobutyric acid (mM)	2.38	2.28	2.09	2.79	2.52	0.089	< 0.001	< 0.001	0.24	0.05
Isovaleric acid (mM)	1.94	1.85	1.64	2.21	2.03	0.091	< 0.001	< 0.001	0.02	0.08
Valeric acid (mM)	1.48	1.41	1.32	1.56	1.6	0.034	< 0.001	0.03	0.04	0.64
Acetic acid/ Propionic acid	4.22	4.23	4.33	4.18	3.64	0.040	0.10	0.03	0.02	< 0.01

^a^Values are average concentrations at 24 and 48 h of incubation. No treatment × time interaction was observed

^b^Average of SEM among treatments

^c^*PM-L* linear effect of pomegranate, *PM-Q* quadratic effect of pomegranate, *PM-C* cubic effect of pomegranate

^d^*Mls* control vs. molasses

The molasses treatment had higher (P 0.01) ADMD, TDMD, TOMD, and MN, but lower (*P* < 0.01) CH_4_ production (Table 5). Propionate, butyrate, and isobutyric levels increased (*P* < 0.05), but the acetate/propionate ratio decreased significantly in the molasses treatment compared to the control (Table 6). With time incubation, the NH_3_-N concentration in molasses was lower than the control (Fig. 2).

### Correlation analysis

**Fig. 3:**
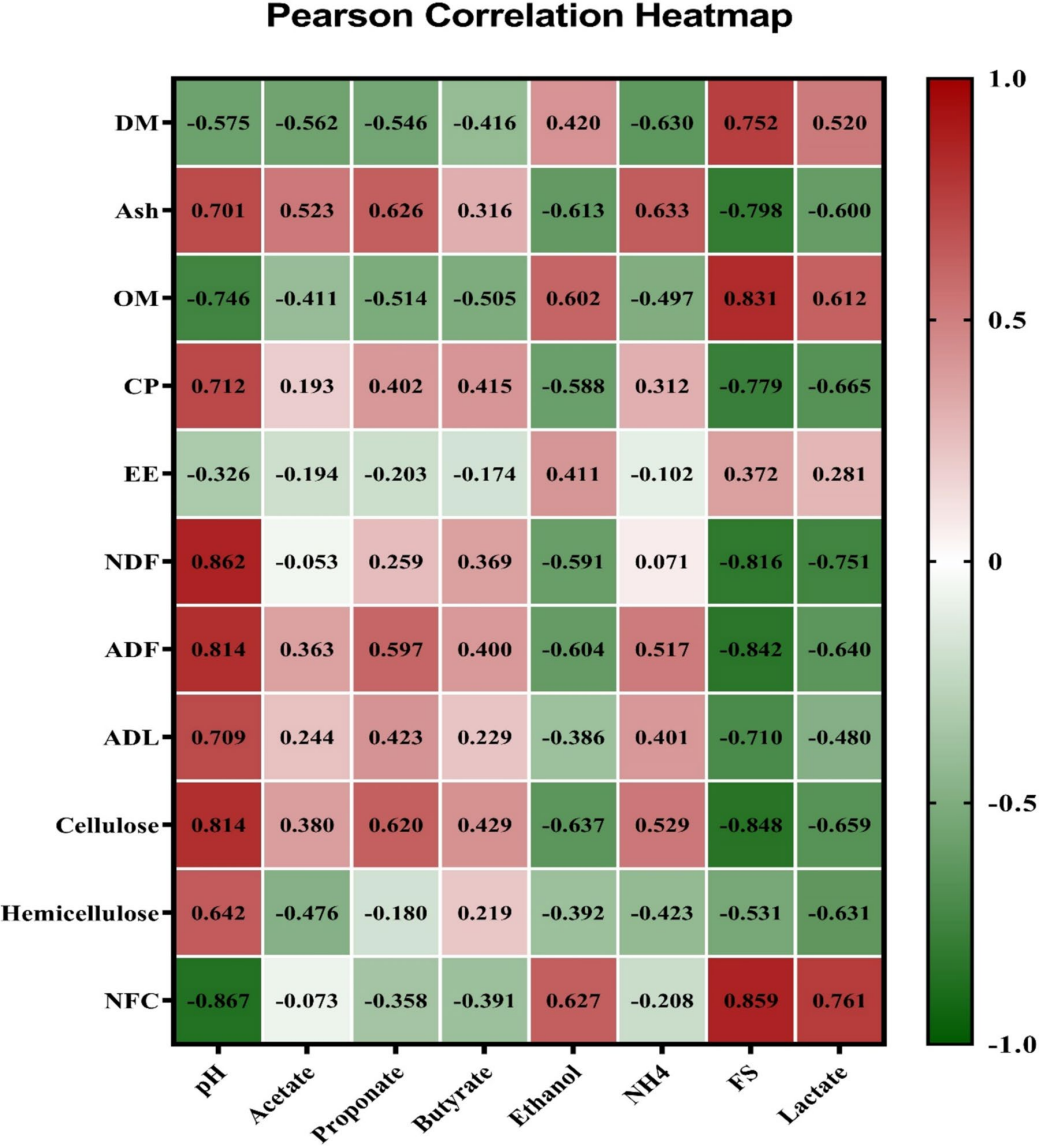
Pearson correlations between the silage quality parameters and chemical composition of berseem silages throughout the ensiling period. DM: dry matter; OM: organic matter; CP: crude protein; EE: ether extract; NDF: neutral detergent fiber; ADL: acid detergent lignin; NFCM: non-fibrous carbohydrates; FS: Fleig’ score. The number in each square represents the correlation extent; the color represents a significant correlation (*p* < 0.05), the deeper the color of the square is the more significant the correlation. the red color means a positive correlation, and the green color means a negative correlation

**Fig. 4:**
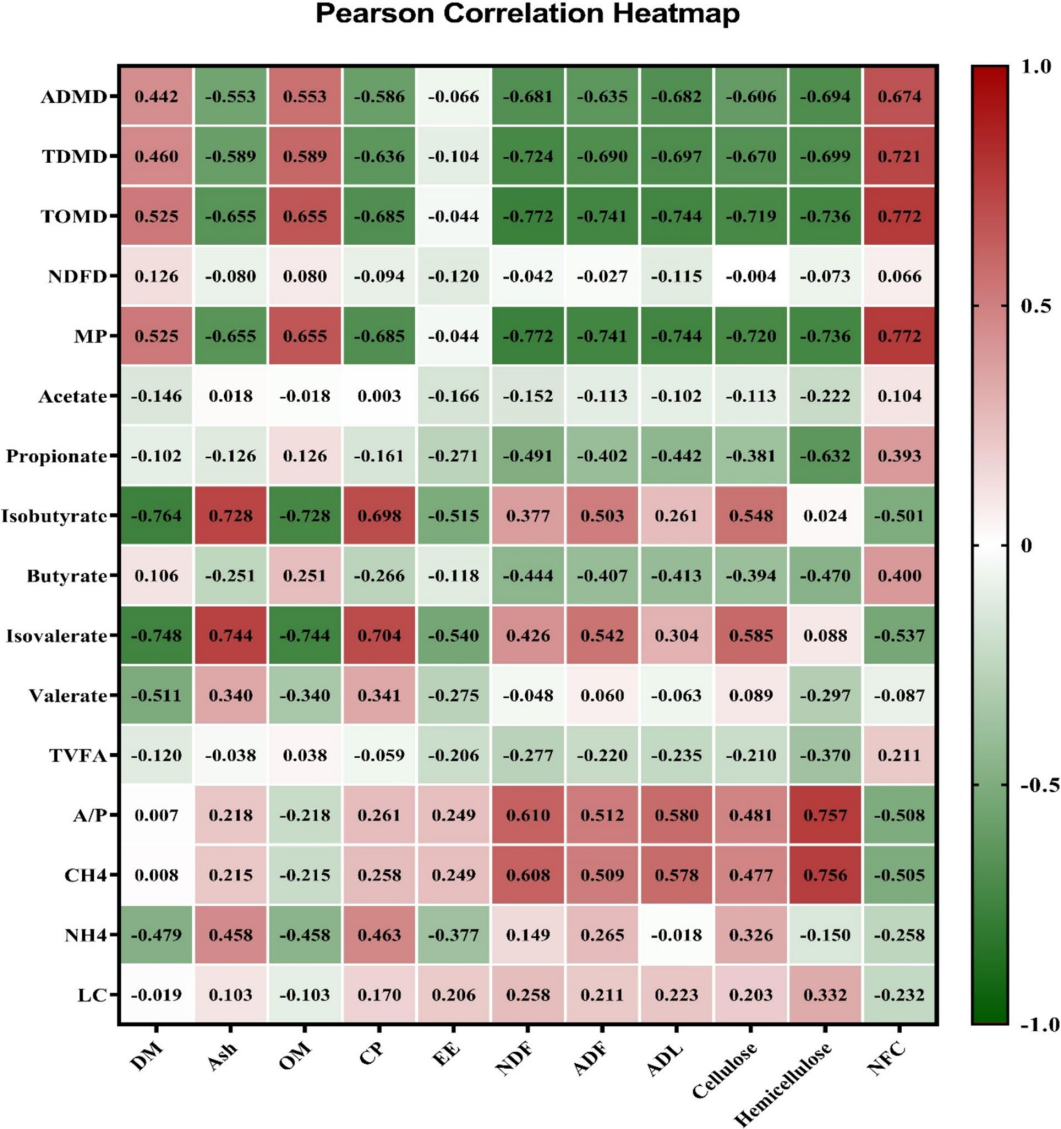
Pearson correlations between the in vitro fermentation parameters at 24 h of incubation and chemical composition of berseem silages. ADMD: apparent dry matter degradability; TDMD: truly dry matter degradability; TDOM: truly degraded organic matter; NDFD: neutral detergent fiber degradability; MP: microbial protein; TVFA: total volatile fatty acid; A/P: acetate to propionate ratio; CH_4_: methane. DM: dry matter; OM: organic matter; CP: crude protein; EE: ether extract; NDF: neutral detergent fiber; ADF: acid detergent fiber; ADL: acid detergent lignin; NFC: non-fibrous carbohydrates. The number in each square represents the correlation extent; the color represents a significant correlation (*p* < 0.05), the deeper the color of the square the more significant the correlation. the red color means a positive correlation, and the green color means a negative correlation

**Fig. 5:**
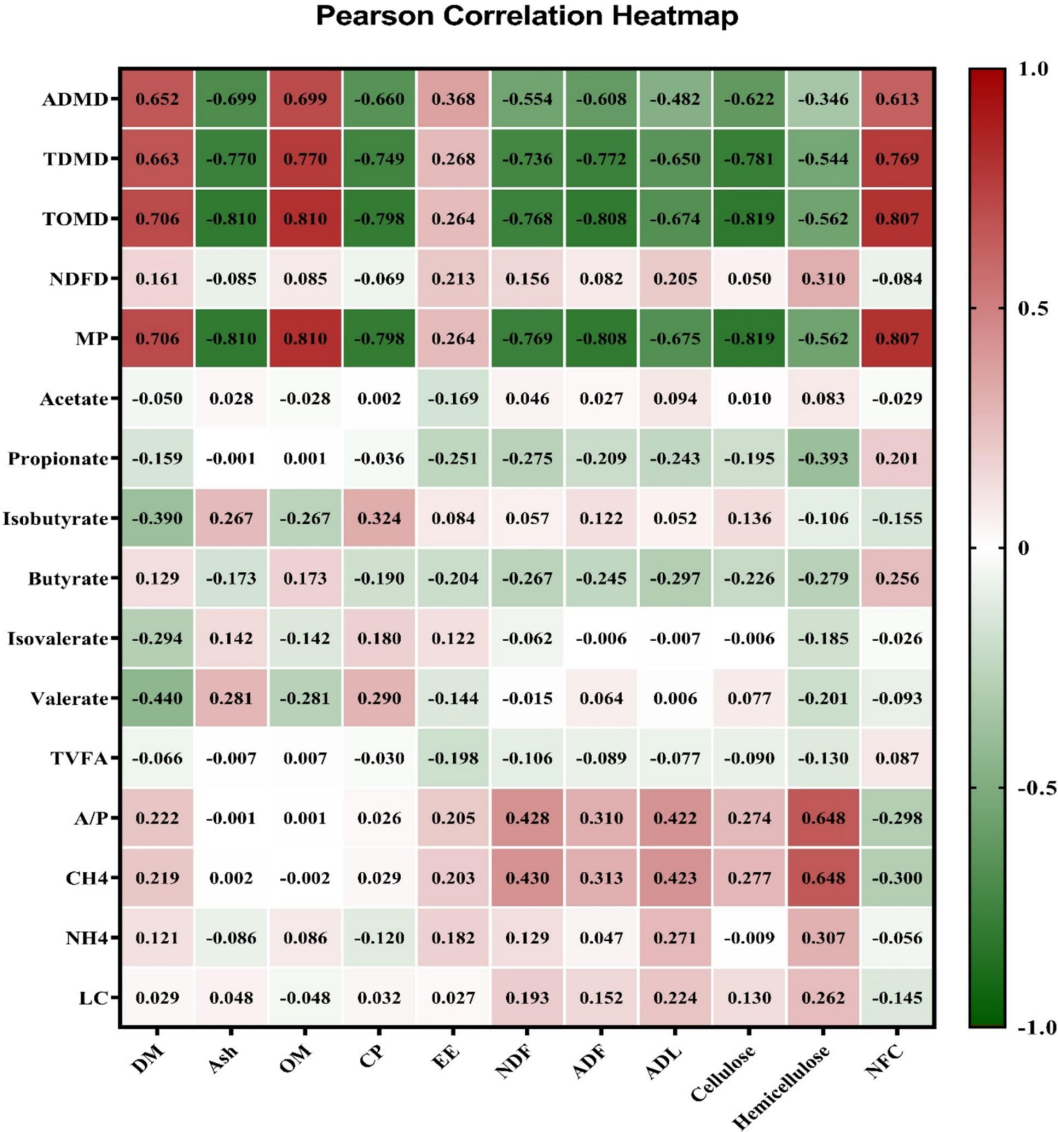
Pearson correlation between the invitro fermentation parameters at 48 h of incubation and chemical composition of berseem silages. ADMD: apparent dry matter degradability; TDMD: truly dry matter degradability; TDOM: truly degraded organic matter; NDFD: neutral detergent fiber degradability; MP: microbial protein: TVFA: total volatile fatty acid; A/P: acetate to propionate ratio; CH_4_: methane. DM: dry matter; OM: organic matter; CP: crude protein; EE: ether extract; NDF: neutral detergent fiber; ADF: acid detergent fiber; ADL: acid detergent lignin; NFC: non-fibrous carbohydrates. The number in each square represents the correlation extent; the color represents a significant correlation (*p* < 0.05), the deeper the color of the square the more significant the correlation. The red color means a positive correlation and the green color means a negative correlation

In EXP 1, the silage pH exhibited positive correlations with CP content (0.712) during the ensiling process, whereas FS and lactate demonstrated negative correlations (−0.779 and −0.665, respectively) with CP content (*P* < 0.01; Fig. 3). A negative correlation was observed between the high NFC concentration and pH (−0.867), while it showed a positive correlation with FS (0.859) and lactate levels (0.761). On the other hand, NDF, ADF, and cellulose exhibited positive correlations with pH (0.862, 0.814, and 0.814, respectively) but negative correlations with FS (−0.816, −0.842, and −0.848, respectively) and lactate (−0.751, −0.640, and −0.659, respectively; *P* < 0.01). In Exp 2, the ADMD, TDMD, and TOMD were negatively associated with nonstructural carbohydrates and positively associated with NFC content in silage after 24 and 48 h of incubation (*P* < 0.01; Figs. 4 and 5). The contents of CP were found to be positively correlated with isobutyrate (0.698) and isovalerate (0.704) while negatively correlated with MN (−0.685), ADMD (−0.586), TDMD (−0.636), and TOMD (−0.685) after 24 h (*P* < 0.01; Fig. 4).

## Discussion

### Experiment 1

In the current study, the DM content of wilted berseem was significantly lower than the recommended range (300–350 g/kg) for optimal silage quality (Kung et al. 2018). To address this issue, common techniques include the incorporation of dried materials or the addition of additives, aiming to reduce moisture content (Borreani et al. 2018). As expected, the inclusion of dried pomegranate peels in this study may help reduce moisture levels of berseem from 750 to 630 g/kg. In comparison to berseem, dried pomegranate peels would make a good ferment substrate for ensiling due to their high NFC content (607 vs 83.6 g/kg DM), which could support sufficient acid production during the ensiling process (Rooke and Hatfield 2003). The TP, TT, and CT values of dried pomegranate peel were lower than those reported by Natalello et al. (2020) and Niu et al. (2023). These differences may be explained by analytical methods or growth conditions that affect polyphenol concentrations, as reviewed by (Aboagye and Beauchemin 2019). Thus, it is hypothesized that adding dried pomegranate peel to ensiled berseem would accelerate fermentation, reducing extensive proteolysis, preventing the negative effects of undesirable bacteria growth, and reducing rumen protein degradation and CH_4_ production in ruminants fed such silage.

In the current study, the pH values of silages found in this study ranged from 4.30 to 5.00, which are considered suitable for legume silage (Kung et al. 2018). The values of lactic acid ranged from 20 to 68 g/kg, which is recommended for legume silages with a low DM concentration (300–350 g/kg), according to Kung et al. (2018). The high tannin content of dried pomegranate peels (Table 1) may explain the low pH of silage and significant lactic acid production by improving silage antioxidative potential and decreasing berseem silage buffering capacity, promoting the growth of lactic acid bacteria and inhibiting undesirable bacteria (He et al. 2020; Zou et al. 2021). Furthermore, the high content of dried pomegranate peels of NFC, specifically pectin (data not estimated), which acts as a substrate for the growth of lactic acid-producing bacteria (e.g., Leuconostoc), results in higher organic acids and lower silage pH (Gao et al. 2021; Wang et al. 2020a).

The greatest decrease in pH values for all ensiled forages was observed within the first 30 days of ensiling, which agrees with previously reported data for various materials (Ahmed et al. 2023; Gao et al. 2021). Lactic acid bacteria, particularly heterofermentative bacteria, grew quickly and reduced pH during the initial fermentation ensiling times, and then their growth decreased with ensiling time due to decreased WSC or low pH sensitivity, which could explain this (Wang et al. 2020b).

Acetic acid levels in the mixture were found to be lower than the optimal range (10–30 g/kg DM; (Kung et al. 2018). The decrease in acetic acid concentrations could be explained by the presence of tannins that inhibit bacteria producing acetic acid such as enterobacteria (Zou et al. 2021). The energy cost of ensiling dried pomegranate peel with berseem silage was expressed as the production of highly undesirable butyric acid and ethanol. This is consistent with the results of Eliyahu et al. (2015) in pomegranate pulp silage. However, the values of butyric acid and ethanol were less than 5 and 30 g/kg DM, respectively, and were within the recommended ranges (Kung et al. 2018; Muck 2010). The massive fermentation of carbohydrates by yeast, heterofermentative lactic acid bacteria, and *Clostridium tyrobutyricum* during the ensiling process may explain the high levels of lactate, butyrate, and ethanol production in dried pomegranate peel (Eliyahu et al. 2015). The NH_3_-N concentration was less than 5 g/kg TN of dried pomegranate peel, which was significantly lower than the recommended ranges for legume silage (Zhang et al. 2016). This could be explained by tannin-protein complex formation and inhibition of plant protease activity during ensilage (Chen et al. 2021).

Molasses additives improved berseem silage quality, which is consistent with the findings of Gao et al. (2021). The high molasses content of soluble substrates may promote homolactic fermentation, raise lactate concentration, and lower pH, resulting in lower butyric acid and NH_3_-N production as well as high ethanol (Desta et al. 2016; Gao et al. 2021).

The higher DM and NFC content observed with higher ratios of dried pomegranate peels in the mixture can be linked to the chemical composition of dried pomegranate peels. However, the decrease in DM content observed in ensiled forages over time is most likely due to microbial fermentation of silage DM, which results in the decomposition of nutrients into liquids, gases, and VFA (Desta et al. 2016). The fluctuations in CP levels in ensiled forages can be ascribed to the breakdown of diverse protein compositions in the ensiled forages, caused by the interaction between pomegranate peel and berseem. This interaction may affect the speed and degree of fermentation (Rooke and Hatfield 2003).

The higher DM content in molasses silage could be due to the higher DM content of the molasses used, which is consistent with the findings of Desta et al. (2016). Furthermore, the high NFC content in molasses silage with ensiling time may be linked to the acid hydrolysis of structural carbohydrates, which liberates WSC and sugars, or the inhibition of silage microbial growth by reducing pH, which prevents the absorption of fermentable substrates (Desta et al. 2016). Further, the reduction in hemicellulose content of silage-treated molasses could be attributed to organic acid accumulation during forage ensiling (Desta et al. 2016).

### Experiment 2

The higher in vitro degradation of dried pomegranate peels in comparison to berseem silage could be ascribed to the lower content of their respective fiber fractions, a relationship supported by the personal correlation of this study. The study revealed a significant negative correlation (P < 0.01) between ADMD, TDMD, and TOMD with the silage content of NDF (−0.681, −0.724, and −0.772, respectively), ADF (−0.635, −0.690, and −0.741, respectively), and ADL (−0.682, −0.697, and −0.744, respectively). Pomegranate peel silage has a high content of substrates potentially degradable (e.g., pectin) (data not estimated) by the ruminal microbiota and low-lignin, which has antimicrobial efficacy (Medjekal et al. 2017). The associative impact of combining berseem and pomegranate peel forages may account for the variations in nutrient degradation observed in different forage mixes. The diverse characteristics and chemical components of the forage blend lead to a lack of synchronization in the release of nutrients, which in turn leads to variations in the growth of microbial biomass (Chen et al. 2022).

The dried pomegranate peel was effective in reducing CH_4_ production at higher levels due to tannins, which have the potential to reduce CH_4_ production, according to Chen et al. (2021). Furthermore, increased microbial protein synthesis plays a significant role in mitigating methanogenesis by creating a competitive environment for hydrogen ions (H^+^), which are required for CH_4_ formation. This competition can successfully reduce CH_4_ emissions (Czerkawski 1986). Pomegranate peel was effective in improving the efficiency of rumen microbial N synthesis, which could be attributed to tannin having antiprotozoal properties (data not estimated) that lead to ammonia reduction, which could be due to decreased bacterial lysosome activity or an increase in NH_3_-N uptake for microbial protein biomass synthesis (Abarghuei and Salem 2021). The substantial amounts of dried pomegranate peel shift VFA molar proportions like monensin (i.e., decrease the acetate and propionate ratio), which agrees with Khorsandi et al. (2019). The decrease in BCVFA levels can be attributed to the high tannin content of pomegranate peels, which is consistent with the findings of Chen et al. (2021) on alfalfa treated with tannic acid. Additionally, the diminished accumulation of BCVFA is associated with a lower concentration of NH_3_-N (Fig. 2). This could be explained by the formation of a tannin and protein complex via hydrogen bonding, as well as the tannins’ inhibitory effects on rumen proteolytic bacteria (Makkar et al. 1993; Ahmed et al. 2024).

In our study, molasses was used as a positive control when adding pomegranate peels to the silage. For ensiling, molasses is a well-known ingredient that works especially well with forages like Berseem that have WSC content. Molasses additions are used as stimulants to increase the availability of fermentable carbohydrates and promote the growth of lactic acid bacteria to improve the quality of fermentation (Li et al. 2010; Gao et al. 2021). Molasses added to silage modifies rumen fermentation profiles, which is consistent with the findings of Chen et al. (2016). This could be due to sucrose’s high molasses content, which serves as an important substrate for microbial fermentation, improving rumen microbial protein synthesis, nutrient degradation, and mitigation of CH_4_ (Chen et al. 2016; Palmonari et al. 2023). The low NH_3_-N concentration of molasses-treated silage could be attributed to the silage’s reduced protein fraction, which should result in a lower protein fraction (Pámanes-Carrasco et al. 2023).

## Conclusions

Both dried pomegranate peel and molasses with berseem as carbohydrate sources were found to be excellent cleaners for the environment and animal nutrition after 30 days of ensiling, with higher fermentation quality and a suitable nutritional composition. In addition, adding dried pomegranate peel and molasses to silage improved nutrient degradation and microbial protein synthesis, reduced ruminal protein degradation, and supported the reduction of CH_4_. As a result, these additives are thought to be safe for both animals and humans when used as antibiotic alternatives in ruminant diets. More in-vivo research is required to corroborate our in-vitro findings.

## Data Availability

The data that support the findings of this study are available from the corresponding author upon reasonable request.
